# Tuberculosis active case finding in Cambodia: a pragmatic, cost-effectiveness comparison of three implementation models

**DOI:** 10.1186/s12879-017-2670-8

**Published:** 2017-08-22

**Authors:** Richard James, Keovathanak Khim, Lydia Boudarene, Joanne Yoong, Chea Phalla, Saly Saint, Pichenda Koeut, Tan Eang Mao, Richard Coker, Mishal Sameer Khan

**Affiliations:** 1 Saw Swee Hock School of Public Health, National University of Singapore, Singapore, Singapore; 2 University of Health Science, Phnom Penh, Cambodia; 3 Center for Economic and Social Research, University of Southern California, 635 Downey Way, VPD, Los Angeles, CA 90089 USA; 4 National Center for Tuberculosis and Leprosy Control, Phnom Penh, Cambodia; 5 Communicable Diseases Policy Research Group, London School of Hygiene & Tropical Medicine, Bangkok, Thailand; 6 Faculty of Public Health, Mahidol University, Bangkok, Thailand

**Keywords:** Tuberculosis, Cambodia, Cost-effectiveness, Case finding

## Abstract

**Background:**

Globally, almost 40% of tuberculosis (TB) patients remain undiagnosed, and those that are diagnosed often experience prolonged delays before initiating correct treatment, leading to ongoing transmission. While there is a push for active case finding (ACF) to improve early detection and treatment of TB, there is extremely limited evidence about the relative cost-effectiveness of different ACF implementation models. Cambodia presents a unique opportunity for addressing this gap in evidence as ACF has been implemented using different models, but no comparisons have been conducted. The objective of our study is to contribute to knowledge and methodology on comparing cost-effectiveness of alternative ACF implementation models from the health service perspective, using programmatic data, in order to inform national policy and practice.

**Methods:**

We retrospectively compared three distinct ACF implementation models - door to door symptom screening in urban slums, checking contacts of TB patients, and door to door symptom screening focusing on rural populations aged above 55 - in terms of the number of new bacteriologically-positive pulmonary TB cases diagnosed and the cost of implementation assuming activities are conducted by the national TB program of Cambodia. We calculated the cost per additional case detected using the alternative ACF models.

**Results:**

Our analysis, which is the first of its kind for TB, revealed that the ACF model based on door to door screening in poor urban areas of Phnom Penh was the most cost-effective (249 USD per case detected, 737 cases diagnosed), followed by the model based on testing contacts of TB patients (308 USD per case detected, 807 cases diagnosed), and symptomatic screening of older rural populations (316 USD per case detected, 397 cases diagnosed).

**Conclusions:**

Our study provides new evidence on the relative effectiveness and economics of three implementation models for enhanced TB case finding, in line with calls for data from ‘routine conditions’ to be included in disease control program strategic planning. Such cost-effectiveness comparisons are essential to inform resource allocation decisions of national policy makers in resource constraint settings. We applied a novel, pragmatic methodological approach, which was designed to provide results that are directly relevant to policy makers, costing the interventions from Cambodia’s national TB program’s perspective and using case finding data from implementation activities, rather than experimental settings.

## Background

Tuberculosis (TB) control remains a critical global challenge, becoming the leading cause of death from an infectious disease in 2014. TB principally affects adults in lower income countries, with 10.4 million new cases estimated in 2015, of which more than half occurred in people living in South-East Asia and Western Pacific regions [1]. The Directly Observed Therapy Short-course (DOTS) strategy to control TB was launched by the World Health Organisation (WHO) in the early 1990’s and was based on the premise that early detection and treatment of new cases would prevent spread and reduce the burden of disease. Global targets for the proportion of new TB cases detected and treated successfully under DOTS were set at 70 and 85% respectively. Despite decades of investment towards these targets and 100% DOTS coverage by 2004 [2], early case finding has been a key challenge for TB program implementers. Currently, an estimated 37% of TB patients globally remain undiagnosed or are not reported [1], and prolonged delays to diagnosis are reported among some patients who do eventually access DOTS services. In recent years, the WHO has raised concerns that the impact of current interventions to improve early detection of TB may have been saturated [3].

It is indeed likely that the current DOTS passive case finding (PCF) strategy, which relies on patients presenting to the health facility for screening of their own accord, has limitations in contexts where primary health services are weak and awareness of TB is low; evidence from a multi-country analysis indicated that DOTS programs may have no impact on improving case detection [4]. PCF approaches may be resulting in both missed diagnoses and delayed diagnosis, leading to transmission of infection, which would be consistent with the small (1–2% per annum) observed annual decline in incidence of TB [5, 6]. At the current rate of decline in incidence, it will take more than 150 years to meet the WHO targets of reducing TB deaths by 95% and incidence by 90%, compared to 2015. In light of this, there is a push for new models of case finding – collectively referred to as Active Case Finding (ACF) approaches- which involve specific out-reach activities by the health system and its staff, in order to identify and test patients who are either symptomatic or considered to be at higher risk of having TB [7, 8]. The advantages of ACF include not only that it potentially identifies TB cases which would not have been diagnosed without the intervention, but may also identify cases at an earlier stage than if relying on PCF alone, thereby reducing transmission [9].

Although referred to using the same terms, numerous different ACF implementation models exist, ranging from population-wide screening to targeted case-finding in high-risk groups [10]. Compared to PCF, ACF requires higher investment and more human resources from National TB Programs, which is a major concern in resource-constrained settings [9]. Reviews of studies have shown that the mode of implementation of TB control activities has a large impact on cost-effectiveness, and researchers have highlighted the need for comparisons of different health service delivery models to inform planning and expansion of programmatic approaches to TB control. This is particularly true for ACF interventions. While globally there is some research comparing ACF implementation models and yields [11, 12], with a study in Zimbabwe finding that 77% of symptomatic, smear-positive study participants had their first investigation for TB through community-based ACF [12], there is very limited information about relative cost-effectiveness of different ACF strategies. A review of over one hundred ACF publications found that no studies conducted a cost effectiveness analysis using established guidelines [13].

Cambodia presents a unique opportunity for addressing this gap in evidence on relative cost-effectiveness of alternative ACF implementation models. The country has one of the highest TB incidence rates in the region, at 380 per 100,000 population and approximately 40% of smear-positive patients remain undiagnosed [2]. Many different ACF interventions have been piloted in Cambodia since 2010 [14–17], and studies thus far have only looked at the cost effectiveness of a single ACF strategies [15, 18]. The National Center for Tuberculosis and Leprosy Control in Cambodia (CENAT), which is the government body responsible for resource allocation decisions on TB control activities, highlighted the urgent need to compare ACF strategies in order to assess which models identify the largest number of new TB cases per unit of investment. In close collaboration with, and in order to inform, CENAT policy makers, we conducted a pragmatic comparison of the cost effectiveness of three large-scale ACF programs from a health system perspective. These programs have been piloted in Cambodia and decisions on scale up need to be made.

## Methods

We compared three ACF programs using retrospective data on case-finding during the implementation period of the pilot program, and primary data collected on costs of implementation, as described below.

### Selection of ACF models for comparison

To initiate the cost-effectiveness comparison, we first compiled a list of all ACF interventions implemented in Cambodia over the past 5 years, through discussions with officials at CENAT and the Cambodia WHO office which had held a meeting of all ACF implementing agencies, supplemented with searches of peer reviewed literature, grey literature and organizational websites. We identified nine different approaches to delivering ACF services across the country by CENAT and various non-governmental organisations (NGOs): symptom screening in prisons; testing contacts of TB patients; testing patients with HIV; door to door symptom screening in urban slums; symptom screening focusing on elderly members of the community; and using community based volunteers for identification of symptomatic patients in rural areas.

For this study, we selected ACF interventions meeting the following criteria, to allow for a comparison across implementation approaches: large-scale (covering more than three districts), primarily targeting adults in the community, seeking to diagnose bacteriologically confirmed (B+) cases using smear-microscopy or Xpert MTB/RIF (GeneXpert) and implemented for a similar time period (10–14 months). We identified three distinct ACF interventions implemented by different public health organisations for inclusion in the analysis. Implementation approaches applied to deliver ACF services are summarized below and in Table 1. All interventions were initiated within a similar timeframe (2012–2013) and were funded through the same mechanism (TB REACH). They therefore followed similar reporting and compliance requirements and were independently assessed by an outside agency selected by the funder [19, 20].

**Table 1:** Summary of TB Active Case Finding (ACF) strategies

	CENAT	HOPE	CATA
Target groups	Neighbourhood and household contacts of TB cases (registered within the last 2 years)	Populations in poor urban settlements, Phnom Penh	Older age (>55 yrs. old) and vulnerable groups
Key activities	• Community health worker visited homes of TB patients registered within last 2 yrs.	• TB-officers and village-health-volunteers performed door to door symptom screening of 315,874 people	• Door to door screening of 68,486 people, focusing on older patients
	• 33,029 household contacts and ‘neighbourhood contacts’ screened using CXR	• 12,201 symptomatic patients identified	• 11,995 symptomatic patients referred for testing and provided transport costs if required
	• 3632 patients with abnormal CXR had a GeneXpert sputum test	• 10,301 symptomatic patients tested with (LED fluorescence) microscopy	• 11,650 underwent CXR screening
		• 1894 patient underwent GeneXpert testing	• 2518 had GeneXpert testing
Operational Districts	15	4	5

### Implementation aspects of ACF models compared

1. Screening contacts of index cases (CENAT) [15]

The national TB program in Cambodia, CENAT conducted an ACF program for 10 months (2012–2013) in 15 operational districts (ODs), using a neighborhood and household contact tracing strategy. Any smear-positive TB cases registered for treatment during the preceding 2 years were located and their household contacts were referred for chest X-Ray (CXR) testing irrespective of presence or absence of symptoms. In addition, people living in the neighbourhood of a confirmed TB case who demonstrated symptoms consistent with TB were referred for the same CXR testing. Community health workers, known as Village Health Support Group (VHSG), were responsible for referring individuals for CXRs. Any person with an abnormal CXR or with persistent symptoms suggestive of TB and a normal CXR underwent GeneXpert sputum testing alone in order to identify B+ TB cases.

(2) Screening of urban poor (HOPE) [14]

Sihanouk Hospital of Hope (HOPE) conducted ACF over a 14-month period (2012–2013), covering four ODs within Phnom Penh city. They targeted poor urban communities, which had a presumed higher prevalence of TB related to socioeconomic factors and reduced access to TB treatment. VHSG, managed by salaried ‘TB workers’, conducted verbal door to door symptom screening and sputum samples were requested from those reporting TB symptoms. Sputum samples were transported for smear-microscopy testing at referral hospitals and the HOPE hospital in Phnom Penh. GeneXpert and sputum culture were utilized when subjects were suspected of having HIV co-infection, being at higher risk of multi drug-resistant (MDR) TB and in those who were negative on microscopy but had persisting symptoms.

(3) Targeted screening in older rural population (CATA)

The Cambodia Anti-Tuberculosis Association (CATA) organisation conducted a 12 month ACF program (2013–2014) covering five ODs. They focused, non-exclusively, on people aged 55 years or older, due to the known higher TB case notification rates in this age group [21]. CATA staff and OD TB focal persons worked with health center staff and VHSG to conduct verbal symptom screening and coordinate CXR and GeneXpert testing in mobile units. Symptomatic patients attending screening at the health centres initially had CXRs and when features suggestive of TB were found, underwent GeneXpert testing. Patients identified with symptoms in remote areas who could not easily visit health centres were tested directly with GeneXpert.

### Calculation of effectiveness

We defined the effectiveness of ACF interventions in terms of the number of new, adult TB cases diagnosed. To allow a comparison between interventions, we applied a standard definition to assess the number of new cases diagnosed: confirmed B+ cases based on smear-microscopy or GeneXpert testing, pulmonary TB, aged 15 or above.

We extracted data from internal reports, published papers, as well the raw data on case finding activities from each program. Published research papers were available for two of the ACF strategies (HOPE [14] and CENAT [15]); the authors were contacted and they provided further reports as well as raw data for analysis. The unpublished reports examined included internal project narrative documents and results data, standardized TB REACH annual reports for each intervention and internal budget information. Discussions were held with implementing staff for each intervention in person and on the phone to fully understand the implementation approach, before and after the data extraction process.

A standardized data extraction tool was used to extract data about the duration of activities, area of coverage and number of eligible B+ cases diagnosed by ACF intervention.

### Costing of ACF implementation activities

For each ACF project, we gathered detailed information about the activities conducted to diagnose new TB cases, including trainings and preliminary meetings prior to initiating ACF activities, in order to estimate costs of implementing the same activities from the health service (CENAT’s) perspective. First, interviews were conducted with key implementation staff involved in the ACF activities of each organization (two per organization) to understand resources used, applying a standardised data collection template (Table 2); this was supplemented with data from official records and reports (including TB REACH yearly reports, internal reports and budgets). After resource information was gathered, follow up interviews were conducted with key personnel from each organisation to clarify and review the resourcing information compiled.

**Table 2:** Breakdown of cost categories used to analyse the ACF strategies

Costing Category	Description
Staff salaries	Staff hired specifically for the project.
Per diem payments	All per diems payments for project related scoping, training and field trips. Usually per diems cover all food, accommodation and travel and CENAT supply a lower rate for travel local within Phnom Penh than overnight stays away.
Trainings and meetings	All training sessions and meetings required for the ACF program, including room hire, materials and preparation.
Equipment	Equipment for each program was listed and compared to the resources CENAT currently has access to; any additional equipment (eg: generators) was costed for.
Transport	Staff, patients or sputum sample transport related to diagnosis of TB.
Consumables	Consumables included items such as petrol, testing materials (X-ray film, GeneXpert cartridges, microscopy reagents) and mobile phone credit.

We then shared data on all the resources required by each ACF intervention with CENAT, who supplied their cost estimates for each type of resource, according to their budget predictions for the coming year (2016), this method, along with the exclusive use of USD dollars, avoided any need to adjust figures according to historic inflation or exchange rates, respectively.

Once we had a unit cost for all of the resources used and activities conducted we calculated the overall costs for each of the three strategies, from the perspective that activities would be conducted by CENAT as part of their programmatic strategy for TB control. If there were additional activities or trainings that CENAT would insist on as part of any ACF strategy they conducted - for example, training relevant staff on GeneXpert equipment - this was added to the budget for HOPE and CATA’s activities.

In addition to the costs incurred during the data collection period, the cost of all trainings and preliminary meetings for each program were calculated and included. The preparation and post data collection activities were not included as each organisation took variable lengths of time with overlapping resources for other activities, making the calculation of accurate times and costs for each activity difficult.

### Cost effectiveness comparison

We sought to estimate the cost per additional B+ case diagnosed for the three different ACF strategies, using a health system cost perspective based on the assumption that the ACF strategies would be implemented by CENAT. We assessed cost-effectiveness using the standard approach used by researchers and funders such as TB REACH, defining the cost per TB case detected as the total cost of activities related to diagnosis divided by the total number of TB cases diagnosed [22].

### Assumptions

The method of analysis employed in this study assumes that the effectiveness achieved for each dollar spent is comparable between the programs, meaning there is an assumption of equivalent efficiency across the three implementing groups. A further assumption made in the comparison, is that the cases detected would not have been detected had the ACF activities not taken place. It should be noted that due to the different contexts of each strategy the consequences of this assumption is likely to vary between the programs.

## Results

Our comparison of three distinct ACF models in Cambodia revealed that the cost per case detected was 249 USD, 308 USD and 316 USD, for the HOPE, CENAT and CATA ACF models, respectively (Table 3). HOPE’s program, working in poor urban areas of Phnom Penh, was the most cost-effective of the three, by a small margin, followed by the model of testing contacts of TB patients (CENAT) and the CATA model focusing on older rural populations.

**Table 3:** Cost effectiveness summary

	CENAT	HOPE	CATA
Intervention months [dates]	10 [Feb 2012 – Dec 2012]	14 [Feb 2012 – Mar 2013]	12 [Mar 2013 – Mar 2014]
Total B+ patients diagnosed	807	737	397
B+ patients diagnosed per month	80.7	52.6	33.1
Total cost (USD)	248,222	183,180	125,297
Cost per month (USD)	24,822	15,265	17,899
Cost per new B+ case diagnosed (USD)	308	249	316

CENAT’s ACF activities involved providing CXRs to 33,029 people and diagnosed 807 B+ patients. HOPE tested 10,301 symptomatic individuals using sputum microscopy and 1894 with GeneXpert, leading to 737 confirmed B+ cases. Finally, CATA focused on older rural subjects, providing 11,650 CXRs after verbal symptom screening with 2518 going onto have GeneXpert testing, leading to 397 confirmed B+ cases.

The costing analysis, summarized in Table 4, showed that the contact tracing ACF model implemented by CENAT had a total cost of USD 248,222, while HOPE and CATA’s models cost 183,180 and 125,297 respectively. Distribution of costs varied between ACF models, with HOPE spending a higher proportion on salaries than the other organizations, while CATA and CENAT spent more on consumables. HOPE used some of its budget on equipment not routinely supplied by CENAT, which included mobile phones for their TB workers and cool boxes for sputum transportation.

**Table 4:** Cost breakdown of each ACF intervention

Budget Categories	CENAT	HOPE	CATA
Staff salaries	32,700 (13)	85,060 (46)	19,880 (16)
Per diem payments	22,572 (9)	31,112 (17)	35,420 (28)
Trainings and meetings	61,474 (25)	20,255 (11)	11,502 (9)
Equipment	0 (0)	1360 (1)	0 (0)
Transport	840 (0)	10,940 (6)	3185 (3)
Consumables	130,636 (53)	34,453 (19)	55,310 (44)
TOTAL /USD	248,222	183,180	125,297

## Discussion

The results show that despite differences in implementation models and populations, cost-effectiveness of the three interventions was relatively similar, with HOPE being the most cost-effective at less than 250 USD per new case diagnosed. The two rural programs of CENAT and CATA, which applied more costly diagnostic protocols involving chest x-rays, had very similar cost-effectiveness results, at USD 308 and 316 per case detected respectively.

Published ACF costs, per case detected, show that a wide range exists globally, from USD 108 in Cambodia (2010) [18] to USD 444–1493 in South Africa (2015) [23] and USD 1581–4000 in Russia (1995) [24]. TB REACH guidance suggests that the cost per additional case (those TB cases detected by the intervention above the local historical trend) detected and treated, for interventions they fund should be up to USD 350, however, this is a different measure to the costing in our study [1, 22].

Increased case detection and high treatment success rates are the cornerstones of global TB control policy [1] and as one of the top 22 high burden countries, Cambodia has implemented multiple strategies to improve its case detection rate. This is the first cost-effective analysis directly comparing alternative models of ACF in Cambodia, and, to our knowledge, no similar comparison has been conducted in other high TB burden countries.

Our study provides timely information in light of calls for cost-effectiveness data from ‘routine conditions’ to be included when programmatic recommendations for TB diagnosis are made [25]. Such cost-effectiveness information is essential to inform resource allocation decisions of national policy makers. Our method of analyzing the three ACF strategies was designed to provide data that is directly relevant to policy makers, taking a pragmatic approach towards costing the interventions from Cambodia’s national TB program’s (CENAT’s) perspective and using case finding data from program activities, rather than experimental settings. It is important to consider that our analysis, by design, compares heterogeneous ACF models with different population characteristics, the most important being a likely variable TB prevalence [22]. Different screening algorithms were used and the urban poor screening by HOPE used cheaper diagnostic techniques compared to the other two; thus the logistics and costs of each project varied. HOPE also focused exclusively on urban areas where the higher population density meant that larger numbers of people could be screened within a smaller geographical area.

A potential limitation relates to the estimation of costs by CENAT and an assumption that scale up of CATA and HOPE strategies by CENAT will be possible and provide the same efficiency, without substantial extra investment in the current services and health centres. However, there were also advantages to the method employed for cost calculations, with the unit costs being standardized according to national program estimates. We acknowledge that reductions in patient costs owing to outreach activities are not accounted for, and that the cost-effectiveness comparison does not consider equity and accessing of hard to reach populations, which may justify a higher cost per case detected. Finally, we recognize that wider questions regarding ACF itself still remain and highlight that ACF strategies will only be effective if cases are identified early such that onward transmission is prevented. Thus, before launching into large ACF programs in resource-limited settings, pragmatic comparisons of different ACF models – looking not only at cost per case detected as we have done but also at forecasts of long term impact on TB incidence- are required to inform resource allocation decisions.

## Conclusion

Alternative ACF implementation models for TB are being introduced in numerous resource-constraint settings, but evidence on relative cost-effectiveness, which is essential to inform resource allocation decisions of national policy makers, is lacking. We applied a novel, pragmatic methodological approach to compare the cost-effectiveness of three ACF implementation models that had been implemented between 2012 and 2013 in Cambodia, allowing the models to be ranked in terms of potential to identify the largest number of new bacteriologically-positive TB cases per unit of investment. We found that implementation of ACF activities in urban areas was slightly more cost-effective than in rural areas, and that the pragmatic cost-effectiveness assessment approach provided information that was called for by local policy makers.

## Abbreviations

ACF: Active case finding
CATA: The Cambodia Anti-Tuberculosis Association
CENAT: The National Center for Tuberculosis and Leprosy Control in Cambodia
CXR: Chest X-Ray
DOTS: Directly Observed Therapy Short-course
HOPE: Sihanouk Hospital of Hope
MDR TB: Multi drug-resistant tuberculosis
NECHR: The National Ethic Committee for Health Research
NGO: Non-governmental organisations
ODs: Operational districts
PSF: Passive case finding
TB: Tuberculosis
VHSG: Village Health Support Group
WHO: World Health Organisation
